# Molecular dissection of human B-cell tolerance – insights from patients with rare genetic diseases

**DOI:** 10.1186/2194-7791-1-S1-A16

**Published:** 2014-09-11

**Authors:** Henner Morbach, Greta Meyers, Yen-Shing Ng, Jean-Nicolas Schickel, Laurence Menard, Sergei Rudchenko, Jessica L Rojas, Charlotte Cunningham-Rundles, Mary Ellen Conley, Ismail Reisli, Jose Luis Franco, Eric Meffre

**Affiliations:** University Children’s Hospital, Würzburg, Germany; Department of Immunobiology, Yale University School of Medicine, New Haven, CT USA; Laboratory of Biochemistry and Molecular Immunology, Hospital for Special Surgery, New York, NY USA; Group of Primary Immunodeficiencies, University of Antioquia, Medellín, Colombia; Department of Medicine, Mount Sinai Medical Center, New York, NY USA; Department of Pediatrics, University of Tennessee College of Medicine, Memphis, TN USA; Department of Immunology and Allergy, Meram Medical Faculty, Selcuk University, Konya, Turkey

B cells play a central role in the pathogenesis of many autoimmune diseases. Therefore, understanding the mechanisms that regulate B-cell tolerance in humans is important for the development of new therapeutic strategies. Patients with monogenic diseases provide rare opportunities to study the impact of specific gene mutations on the regulation of human B cell tolerance. By this, we could show that alterations in B-cell receptor and Toll-like receptor (TLR) signaling pathways result in defective central B-cell tolerance.

To further dissect the signaling pathways involved in the establishment of central B-cell tolerance in humans, we tested by ELISA and immunofluorescence the reactivity of recombinant antibodies cloned from single transitional B cells from individuals carrying *CD19* mutations. CD19 is a co-receptor expressed on B cells and is involved in the amplification of B-cell responses.

We found that individuals carrying *CD19* mutations displayed defective central B-cell tolerance checkpoints. In addition, CD19-deficient transitional B cells were enriched in anti-nuclear clones, a feature previously observed in IRAK4- and MYD88-deficient patients in which TLR7/9 sensing nucleic acids cannot signal. Therefore, we investigated the functions of these TLRs in B cells in the absence of CD19 expression. CD19-deficient human B cells displayed defective up-regulation of activation markers after TLR7/9 triggering and failed to induce BTK, AKT but not p38 MAPK or Iκ-Bα phosphorylation after TLR7/9 stimulation. Additionally, inhibitors blocking BTK, AKT and PI3K function impaired CD19-dependent TLR7/9 responses in healthy donor’s B cells. Finally, we demonstrated that individuals carrying *BTK* mutations display similar defects in TLR7/9-induced B-cell activation and central B-cell tolerance.

Hence, we identified a previously unsuspected role for CD19 molecules in regulating TLR7/9 functions in human B cells and central B-cell tolerance to nuclear antigens.

